# Evaluation of the NIH Toolbox Odor Identification Test across normal cognition, amnestic mild cognitive impairment, and dementia due to Alzheimer's disease

**DOI:** 10.1002/alz.13426

**Published:** 2023-08-21

**Authors:** Shiloh L. Echevarria‐Cooper, Emily H. Ho, Richard C. Gershon, Sandra Weintraub, Thorsten Kahnt

**Affiliations:** ^1^ Department of Neurology Northwestern University Feinberg School of Medicine Chicago Illinois USA; ^2^ Department of Medical Social Sciences Northwestern University Feinberg School of Medicine Chicago Illinois USA; ^3^ Mesulam Center for Cognitive Neurology and Alzheimer's Disease Northwestern University Feinberg School of Medicine Chicago Illinois USA; ^4^ Department of Psychiatry and Behavioral Sciences Northwestern University Feinberg School of Medicine Chicago Illinois USA; ^5^ Cellular and Neurocomputational Systems Branch National Institute on Drug Abuse Intramural Research Program Baltimore Maryland USA

**Keywords:** aging, Alzheimer's disease, mild cognitive impairment, NIH Toolbox, olfaction

## Abstract

**INTRODUCTION:**

Olfactory decline is associated with cognitive decline in aging, amnestic mild cognitive impairment (aMCI), and amnestic dementia associated with Alzheimer's disease neuropathology (ADd). The National Institutes of Health Toolbox Odor Identification Test (NIHTB‐OIT) may distinguish between these clinical categories.

**METHODS:**

We compared NIHTB‐OIT scores across normal cognition (NC), aMCI, and ADd participants (*N* = 389, ≥65 years) and between participants positive versus negative for AD biomarkers and the *APOE ε*4 allele.

**RESULTS:**

NIHTB‐OIT scores decreased with age (*p* < 0.001) and were lower for aMCI (*p* < 0.001) and ADd (*p* < 0.001) compared to NC participants, correcting for age and sex. The NIHTB‐OIT detects aMCI (ADd) versus NC participants with 49.4% (56.5%) sensitivity and 88.8% (89.5%) specificity. NIHTB‐OIT scores were lower for participants with positive AD biomarkers (*p* < 0.005), but did not differ based on the *APOE ε*4 allele (*p* > 0.05).

**DISCUSSION:**

The NIHTB‐OIT distinguishes clinically aMCI and ADd participants from NC participants.

**Highlights:**

- National Institutes of Health Toolbox Odor Identification Test (NIHTB‐OIT) discriminated normal controls from mild cognitive impairment.
- NIHTB‐OIT discriminated normal controls from Alzheimer's disease dementia.
- Rate of olfactory decline with age was similar across all diagnostic categories.
- NIHTB‐OIT scores were lower in participants with positive Alzheimer's biomarker tests.
- NIHTB‐OIT scores did not differ based on *APOE* genotype.

## 1 BACKGROUND

Alzheimer's disease (AD) is a progressive neurodegenerative disease that affects memory and cognition, leading to reduced independence in activities of daily living and lower life expectancy.^1^ The gradual progression from a state of normal cognition (NC) to dementia of the Alzheimer's type (ADd) involves an intermediary stage termed amnestic mild cognitive impairment (aMCI), wherein memory impairments are present, but not severe enough to impact daily living.^2^ Identifying individuals in the earliest disease stages and at high risk for developing ADd will help target preventative measures and slow disease progression. Recent attention has focused on functional changes in sensory systems, particularly in olfaction, that may either influence or predict aMCI‐ and ADd‐related cognitive impairment.^3^, ^4^

Olfactory decline has been proposed as an early indicator of aMCI and ADd.^3^, ^5^, ^6^ While olfactory decline can occur in the normal aging trajectory,^7^, ^8^, ^9^ this decline may occur earlier and is more severe in individuals who go on to develop symptoms of aMCI and ADd.^10^, ^11^, ^12^, ^13^, ^14^ Estimates suggest that 85% and 90% of individuals with ADd have impaired olfaction,^15^, ^16^ and the severity of olfactory decline correlates with the severity of cognitive impairment.^17^, ^18^ In addition to age, AD risk factors include carrying a copy of the apolipoprotein E (*APOE*) *ε*4 allele,^19^ and preclinical presence of amyloid β and/or tau pathology.^20^, ^21^, ^22^, ^23^ Olfactory deficits correlate with the degree of tau pathology,^24^, ^25^ and occur earlier in life for *APOE ε*4 allele carriers.^26^, ^27^

Typically, brain imaging, extensive cognitive testing, and specialist evaluation are required to clinically diagnose aMCI and ADd.^28^ Evaluating pre‐clinical ADd risk factors using genotyping or testing for known AD biomarkers is time‐consuming and expensive. AD biomarker tests are invasive, involving positron emission tomography (PET) scans with injected radioactive tracers or lumbar spinal taps to evaluate cerebrospinal fluid markers. Currently, blood‐based biomarkers are being developed but are not fully validated.^29^, ^30^, ^31^ Identifying a low‐cost, non‐invasive, and easily interpretable measure that can accurately flag individuals at risk of cognitive decline is thus imperative to facilitating earlier diagnosis. Olfactory identification tests are time‐ and cost‐effective and simple to administer, and may serve to identify these at‐risk individuals so that they may be referred for more in‐depth neuropsychological evaluation and care.

In the present study, we evaluate performance on the National Institutes of Health Toolbox Odor Identification Test (NIHTB‐OIT) ^32^ as part of a multisite study, Advancing Reliable Measurement in Alzheimer's Disease and Cognitive Aging (ARMADA).^33^ The ARMADA study is unique in that it collected data from a population‐based sample across nine study sites in the United States. Many of the ARMADA sites are housed in national Alzheimer's Disease Research Centers (ADRCs) that recruit and longitudinally follow research participants aged 65 and above. The NIHTB‐OIT is a nine‐item multiple choice test administered using scratch‐and‐sniff cards and a tablet. NIHTB‐OIT scores can be easily interpreted by health care workers or trained personnel. We evaluated performance on the test across clinical diagnostic categories designated as NC, aMCI, and ADd, while controlling for age and sex. We then evaluated the test's ability to discriminate between aMCI and NC participants, and between ADd and NC participants. We additionally evaluated NIHTB‐OIT score associations with *APOE ε*4 allele carrier status and with general AD biomarker presence in a subset of participants with available data. Finally, as the NIH Toolbox was initially validated up to age 85, we compared NIHTB‐OIT scores between NC participants aged 65 to 84 and NC participants aged ≥85.

## 2 METHODS

In the present study, we evaluate performance on the NIHTB‐OIT for a general population cohort of adults aged 65 and over, across NC, aMCI, and ADd diagnoses. The dataset used in the present study was obtained through the overarching ARMADA study.^33^ Participants in the ARMADA study were recruited across nine separate study sites, including Northwestern University, University of Michigan, University of Wisconsin‐Madison, Mayo Clinic (Jacksonville, Florida), University of Pittsburgh, Emory University, University of California‐San Diego, Columbia University, and Massachusetts General Hospital. Participants were recruited from existing research cohorts in or affiliated with this network of ADRCs funded by the National Institute on Aging (NIA) and other NIH‐funded longitudinal studies that use similar methods to the ADRC longitudinal studies to clinically classify participants. The ARMADA study was reviewed and approved by the Northwestern University (lead site) Institutional Review Board (IRB #STU00205290). In addition, each of the participating sites also submitted ARMADA for review to their own IRBs and received approval. All research was completed in accordance with the Helsinki Declaration.

Special emphasis was placed on diversity, equity, and inclusion in recruiting participants so that the resulting general population dataset is approximately racially representative of the United States population. Additionally, emphasis was placed on recruiting a group of NC participants over the age of 85 in order to evaluate performance on the NIHTB‐OIT for this age group. Available data through the ARMADA study includes scores on the NIHTB tests,^34^, ^35^ and measures of cognitive functioning, health history, and mental health history through the Uniform Data Set (UDS) procedures adopted by ADRCs.^36^, ^37^ For a subset of participants, *APOE* genotype and/or results from a variety of AD biomarker tests (cerebrospinal fluid [CSF] Aß40/42, total tau, or hosphor‐tau measures, and/or amyloid‐PET imaging) were also available.

### 2.1 NIH Toolbox Odor Identification Test

The NIHTB‐OIT^32^ is a nine‐item test administered to participants by a trained examiner. The test includes nine odors, presented one at a time, on scratch‐and‐sniff cards. For each odor, the participant must choose from four options, including one correct response and three distractors. For each odor, four picture and word options are presented simultaneously in a 2 × 2 layout on a tablet screen and read aloud one at a time by the examiner. Total scores are recorded as integer values ranging from 0 to 9, with chance performance at 25%, roughly a score of 2. The nine target odors that the participant must identify include lemon, Play‐Doh, bubble gum, chocolate, popcorn, coffee, smoke, natural gas, and flower. In the original validation paper,^32^ item‐level accuracy for participants in the 65 to 85 age group ranged from 65% to 92%, with the exception of the Play‐Doh odor (26%). All participants in the present study took the English language version of the NIHTB‐OIT. More information, including an example response screen and examiner training information, can be found on the NIH Toolbox website.^38^

### 2.2 Study participants

Baseline visit data collected from *N* = 389 participants as part of the overarching ARMADA study were included in the present study. Baseline visit data were collected between May 17, 2017, and March 21, 2022, with 96% of the baseline visit data collected prior to January 31, 2020, the start of the COVID‐19 public health emergency in the United States. Participants were included for analysis in the present study if they (1) had completed the NIHTB‐OIT; and (2) had no record in the UDS dataset of previous traumatic brain injury or stroke, both of which may affect olfaction.^39^, ^40^ From the total of *N* = 415 ARMADA study participants who completed the NIHTB‐OIT, *N* = 8 participants were excluded for history of stroke, and *N* = 18 were excluded for history of traumatic brain injury, leaving a total of *N* = 389 participants in the present analysis. Participants were divided into the three research clinical diagnostic categories of NC, aMCI, and ADd. Diagnostic categories had been assigned based on a combination of clinician diagnosis, neurocognitive testing, and the participant's Clinical Dementia Rating (CDR) score.^41^ In the NC group, a special emphasis group of participants aged 85 and older was recruited in order to evaluate performance on the NIHTB‐OIT for individuals aged 85 and older, the maximum age included in the original NIHTB validation study. Out of our total sample, *N* = 124 participants across all diagnostic categories (NC aged ≥85, *N* = 16; NC aged 65 to 84, *N* = 61; aMCI, *N* = 34; and ADd, *N* = 13) returned after 1 year for follow‐up participation in the ARMADA study. These follow‐up data were collected between January 10, 2019, and April 14, 2022 (72% collected prior to January 31, 2020). Since none of these participants changed diagnostic categories, we correlated NIHTB‐OIT scores between their baseline and 1‐year follow‐up visit to evaluate the test‐retest reliability of the NIHTB‐OIT.

**RESEARCH IN CONTEXT**

1. **Systematic review**: We reviewed the literature using databases such as PubMed, revealing that olfactory decline may be an early indicator of cognitive impairment. This study contributes to recent interest in using performance measures to efficiently screen for cognitive impairment.
2. **Interpretation**: As part of the multisite ARMADA (Assessing Reliable Measurement in Alzheimer's Disease and Cognitive Aging) study, we evaluated the NIH Toolbox Odor Identification Test (NIHTB‐OIT) in a cross‐sectional sample of cognitively normal controls and participants with clinical diagnoses of amnestic mild cognitive impairment or Alzheimer's disease dementia. We found that the NIHTB‐OIT differentiates normal controls from the two clinical groups. Participants across groups with AD biomarkers present also had lower scores.
3. **Future directions**: Future studies will evaluate NIHTB‐OIT utility in African‐American and Spanish‐speaking minority cohorts. Additionally, we will assess longitudinal decline on the NIHTB‐OIT in conjunction with Alzheimer's disease progression.

*APOE* genotype data were available for a subset of *N* = 275 participants. Participants were collapsed into two groups, regardless of diagnostic category: an *ε*4 allele‐positive group (at least one *ε*4 allele, *N* = 98) and an *ε*4 allele‐negative group (no *ε*4 allele, *N* = 177). AD biomarker data were also available for a subset of *N* = 165 participants. The availability of specific biomarker tests varied across participants, and included amyloid‐PET scans and/or CSF Aß40/42, total tau, or hosphor‐tau measures. To achieve sufficient statistical power for comparison, participants were collapsed across biomarker tests into two groups, regardless of diagnostic category: an AD biomarker‐positive group (*N* = 48) for participants with biomarker test results consistent with AD, and an AD biomarker‐negative group (*N* = 177) for participants with biomarker test results inconsistent with AD. Positive or negative assignments for the amyloid‐PET scans were determined based on standardized uptake value ratios or distribution volume ratios readings, with cut points at 1.35 and 1.19, respectively. Study sites collecting CSF biomarker measures used varying methods to designate results for each participant as “Consistent with AD” or “Inconsistent with AD.” Details regarding each study site's specific amyloid‐PET methods and CSF biomarker methods are included in table 4 of the original ARMADA project methods paper.^33^

A detailed demographic description of the ARMADA study's baseline visit cohorts (NC aged 65 to 84, aMCI, and ADd) was published in a previous paper.^42^ This previous publication includes detailed information on participant demographics including race and education, recruitment and diagnostic methods, clinical characteristics including CDR scores and the Neuropsychiatric Inventory Questionnaire,^43^ and biomarker and *APOE* data availability across groups. A summary of the demographics, available *APOE* genotype data, and AD biomarker group assignments of participants included in the present study is provided in Table 1.

**TABLE 1:** Participant demographics and available AD biomarker and *APOE* genotype data

Diagnosis	*N*	Sex	Age (mean years ± SD)	AD biomarker data (*N* positive, *N* negative)	> APOE e4 allele data (*N* carriers, *N* con‐carriers)	Reported race	Hispanic/Latino	Study site distribution
Normal cognition, aged ≥85	96	42 Male, 54 Female	87.7 ± 2.1	9, 21	53, 13	White, *N* = 85 Black, *N* = 10 Asian, *N* = 1	Yes, *N* = 1 No, *N* = 95	Emory, *N* = 10 Mass, *N* = 24 Mayo, *N* = 3 NU, *N* = 19 UCSD, *N* = 8 UMich, *N* = 4 UPitt, *N* = 15 UWisc, *N* = 13
Normal cognition, aged 65 to 84	152	50 Male, 102 Female	72.7 ± 5.1	12, 89	57, 29	White, *N* = 123 Black, *N* = 24 American Native, *N* = 2 More than one Race, *N* = 1 Not Provided = 2	Yes, *N* = 3 No, *N* = 149	Emory, *N* = 4 Mass, *N* = 59 Mayo, *N* = 5 NU, *N* = 17 UCSD, *N* = 2 UMich, *N* = 30 UPitt, *N* = 2 UWisc, *N* = 33
Amnestic mild cognitive impairment	79	47 Male, 32 Female	77.3 ± 6.9	14, 5	38, 29	White, *N* = 64 Black, *N* = 15	Yes, *N* = 2 No, *N* = 77	CU, *N* = 4 Emory, *N* = 5 Mass, *N* = 1 NU, *N* = 14 UCSD, *N* = 9 UMich, *N* = 27 UWisc, *N* = 19
Alzheimer's disease dementia	62	34 Male, 28 Female	75.4 ± 7.2	13, 2	29, 27	White, *N* = 55 Black, *N* = 4 Asian, *N* = 1 More than one race, *N* = 1 Not Provided, *N* = 1	Yes, *N* = 2 No, *N* = 60	Emory, *N* = 4 NU, *N* = 10 UCSD, *N* = 5 UMich, *N* = 28 UWisc, *N* = 15
Total	389	173 Male, 216 Female	77.8 ± 8.0	48, 117	177, 98	White, *N* = 327 Black, *N* = 53 Asian, *N* = 2 American Native, *N* = 2 More than one Race, *N* = 2 Not Provided, *N* = 3	Yes, *N* = 8 No, *N* = 381	CU, *N* = 4 Emory, *N* = 23 Mass, *N* = 84 Mayo, *N* = 8 NU, *N* = 60 UCSD, *N* = 24 UMich, *N* = 89 UPitt, *N* = 17 UWisc, *N* = 80

*Note*: Two participants included in the dementia group were below age 65 (aged 62 and 63). Study site abbreviations are as follows: Columbia University, CU; Emory University, Emory; Massachusetts General Hospital, Mass; Mayo Clinic (Jacksonville, Florida), Mayo; Northwestern University, NU; University of California‐San Diego, UCSD; University of Michigan, UMich; University of Pittsburgh, UPitt; University of Wisconsin‐Madison, UWisc.

### 2.3 Statistical analyses and evaluation of the NIHTB‐OIT across diagnostic groups

For the present study, we first calculated NIHTB‐OIT score summary statistics across diagnostic categories. These included the mean, standard deviation, range, N at floor, N at ceiling, skewness, and kurtosis of the distribution of odor identification scores for each group.

We then evaluated NIHTB‐OIT scores using a multiple linear regression model with the equation:

(1) $$\mathrm{NIHTB}-\mathrm{OIT}\;\mathrm{Score}∼\beta_{o}+\mathrm{Age}\beta_{1}+\mathrm{Sex}\beta_{2}+\mathrm{Diagnosis}\beta_{3}.$$

We fit this main‐effects model for the entire participant population (*N* = 389) with the NC aged 65 to 84 and NC aged ≥85 groups collapsed into one NC group in order to characterize performance on the NIHTB‐OIT across the entire sampled age span. With this model, we assessed relationships between olfactory performance, age, and diagnosis, while controlling for sex. We also fit a second model including an interaction term between age and diagnosis, and a third main effects model excluding the NC aged ≥85 from the NC group. For each multiple regression model, age was centered at 77.8 years, the mean age of the entire participant pool.

We then used two logistic regression models to evaluate the NIHTB‐OIT's ability to distinguish between diagnostic categories. The first model evaluated whether odor scores, age, and sex can accurately predict whether a participant is in the aMCI versus the NC aged 65 to 84 group with the following equation:

(2) $$\;\ln\left(\frac{P\left(\mathrm{aMCI}\right)}{1-P\left(\mathrm{aMCI}\right)}\right)=\;\beta_{0}+\;\mathrm{NIHTB}-\mathrm{OIT}\;\mathrm{Score}\beta_{1}+\mathrm{Sex}\beta_{2}+\mathrm{Age}\beta_{3},$$

where *P*(aMCI) refers to the computed probability of having aMCI given odor score, age, and sex. The second model evaluated whether odor scores, age, and sex can accurately predict whether a participant is in the ADd group versus the NC aged 65 to 84 group, with the following equation:

(3) $$\;\ln\left(\frac{P\left(\mathrm{ADd}\right)}{1-P\left(\mathrm{ADd}\right)}\right)=\;\beta_{0}+\;\mathrm{NIHTB}-\mathrm{OIT}\;\mathrm{Score}\beta_{1}+\mathrm{Sex}\beta_{2}+\mathrm{Age}\beta_{3},$$

where *P*(ADd) refers to the computed probability of having ADd given odor score, age, and sex. Participants in the NC aged ≥85 group were not included in the two logistic regression analyses, as they were not properly age‐matched to the aMCI and ADd groups. Overall, there were *N* = 21 participants in the aMCI and ADd groups who were 85 or above, and the mean ages of the aMCI (mean ± standard deviation; 77.3 ± 6.9) and ADd (75.4 ± 7.2) groups were much closer to the mean age of the NC aged 65 to 84 group (72.7 ± 5.1) compared to the NC aged ≥85 group (87.7 ± 2.1).

We plotted two receiver operating characteristic (ROC) curves based on the fitted values of the logistic regression models and calculated the area under the curve (AUC) for each model, indicating how well NIHTB‐OIT scores, age, and sex can distinguish between aMCI and NC aged 65 to 84 participants, and between ADd and NC aged 65 to 84 participants. For each model, we report the fitted probability of having aMCI or AD for each NIHTB‐OIT score, as well as the sensitivity, specificity, positive predictive value, and negative predictive value for each model.

Further, we used two three‐way analyses of covariance (ANCOVAs) to compare differences in odor scores across the two risk‐factor groups: AD biomarker presence (positive vs. negative) and *APOE ε*4 allele carrier status (one or more *ε*4 allele vs. no *ε*4 alleles), controlling for age and sex. Lastly, we used a two‐way ANCOVA to evaluate differences in NIHTB‐OIT scores between NC age groups (aged 65 to 84 vs. aged ≥85), controlling for sex.

## 3 RESULTS

Table 2 displays the summary statistics for the NIHTB‐OIT scores across each clinical cohort. In addition to the baseline scores presented in Table 2, 1‐year follow‐up scores on the NIHTB‐OIT were obtained for *N* = 124 participants, which we used to evaluate the test‐retest reliability of the NIHTB‐OIT. For the entire population (*N* = 124), the correlation between scores at baseline and 1‐year follow‐up visits was *r* = 0.69 (95% confidence interval [CI] 0.59 to 0.78, *p* < 0.0001). The correlation for the NC aged 65 to 84 group (*N* = 61) was *r* = 0.57 (95% CI 0.37 to 0.72, *p* < 0.0001); and the correlation for the aMCI group (*N* = 34) was *r* = 0.71 (95% CI 0.49 to 0.85, *p* < 0.0001). The NC aged ≥85 group (*N* = 16) and the ADd group (*N* = 13) with 1‐year follow‐up visit NIHTB‐OIT scores were too small to compute adequately powered within‐group correlations.

**TABLE 2:** Summary of National Institutes of Health Toolbox Odor Identification Test scores by diagnosis

Diagnosis	*N*	Mean ± SD	Range	*N* at Floor	*N* at Ceiling	Skewness	Kurtosis
Normal cognition (aged ≥85)	96	6.14 ± 1.70	2–9	0	2	−0.73	2.83
Normal cognition (aged 65 to 84)	152	6.97 ± 1.71	2–9	0	33	−0.75	2.94
Amnestic mild cognitive impairment	79	5.32 ± 2.18	0–9	1	4	−0.16	2.14
Alzheimer's disease dementia	62	4.10 ± 2.01	0–9	1	1	0.01	2.45
Total	389	5.97 ± 2.12	0–9	2	40	−0.53	2.53

### 3.1 NIHTB‐OIT scores across age and diagnostic categories

We fit a multiple linear regression model to evaluate the decline of NIHTB‐OIT performance with age and differences in odor scores across diagnostic categories. The model was significant with adjusted *R*2 = 0.30 (*F*_4,384_= 40.85, *p* < 0.001). The resulting model coefficients are displayed in Table 3 (Equation 1). This model suggests that NIHTB‐OIT scores decrease by 0.07 points for every year of increase in age (1 point every 14.3 years). Females scored on average 0.530 ± 0.186 points higher than males across diagnostic categories (*p* < 0.005). Participants in the aMCI group scored on average 1.295 ± 0.235 points lower than NC participants (*p* < 0.001), while those with ADd scored on average 2.675 ± 0.258 points lower than those with NC (*p* < 0.001).

**TABLE 3:** Fitted linear and logistic regression models

Multiple linear regression model^a^: $\mathrm{NIHTB}-\mathrm{OIT}\;\mathrm{Score}∼\beta_{o}+\mathrm{Age}\beta_{1}+\mathrm{Sex}\beta_{2}+\mathrm{Diagnosis}\beta_{3}$					
Coefficient	Estimate	Standard error	*t*‐value	*p*‐value	Interpretation
Intercept					
(Baseline group = Normal Cognition [all ages])	6.37	0.16	38.95	<0.001	A male NC participant at the mean age of 77.8 years is predicted to have an NIHTB‐OIT score of 6.37.
Age (per year)	−0.07	0.011	−6.14	<0.001	NIIHTB‐OIT scores are predicted to decrease by 0.07 points for every year increase in age.
Sex = female	0.53	0.19	2.85	<0.01	Predicted NIHTB‐OIT scores for females are 0.53 points higher than for males.
Diagnosis = aMCI	−1.30	0.24	−5.52	<0.001	Predicted NIHTB‐OIT scores are 1.3 points lower for aMCI participants compared to NC participants.
Diagnosis = ADd	−2.68	0.26	−10.37	<0.001	Predicted NIHTB‐OIT scores are 2.68 points lower for AD participants compared to NC participants.

Normal cognition (aged 65 to 84) vs. amnestic Mild Cognitive Impairment logistic regression model^a^: $\ln\;\left(\frac{P(\mathrm{aMCI})}{1-P(\mathrm{aMCI})}\right)=\beta_{0}+\mathrm{NIHTB}-\mathrm{OIT}\;\mathrm{Score}\beta_{1}+\mathrm{Sex}\beta_{2}+\mathrm{Age}\beta_{3}$					
Coefficient	Estimate	Standard error	z‐value	*p*‐value	Interpretation
Intercept	1.96	0.54	3.66	<0.001	A male at mean age 77.8, with an NIHTB‐OIT score of 0 has 7.1 to 1 odds of having aMCI compared to NC. (odds ratio = 7.1)
Odor score	−0.29	0.085	−3.42	<0.001	For every 1‐point increase in NIHTB‐OIT score, there is a 25.3% decrease in relative risk for having aMCI.
Sex	−0.94	0.32	−2.90	<0.01	Females have a 60.8% decrease in relative risk for having aMCI compared to males.
Age	0.10	0.028	3.64	<0.001	For every 1‐year increase in age there is a 10.8% increase in relative risk for having aMCI within this sample.^‡^

Normal cognition (aged 65 to 84) vs. Alzheimer's Disease dementia logistic regression model^a^: $\ln\left(\frac{P(\mathrm{ADd})}{1-P(\mathrm{ADd})}\right)=\beta_{0}+\mathrm{NIHTB}-\mathrm{OIT}\;\mathrm{Score}\beta_{1}+\mathrm{Sex}\beta_{2}+\mathrm{Age}\beta_{3},$					
Coefficient	Estimate	Standard error	z‐value	*p*‐value	Interpretation
Intercept	3.46	0.62	5.58	<0.001	A male at mean age 77.8, with an NIHTB‐OIT Score of 0 has 31.8 to 1 odds of having ADd compared to NC. (odds ratio = 31.8)
Odor score	−0.72	0.11	−6.55	<0.001	For every 1‐point increase in NIHTB‐OIT Score, there is a 51.3% decrease in relative risk for having ADd.
Sex	−0.46	0.38	−1.21	>0.05	The effect of sex was not significantly associated with risk of having ADd in this sample.^b^
Age	0.01	0.03	0.34	> 0.05	The effect of age was not significantly associated with risk of having ADd in this sample.^b^

Abbreviations: ADd, Alzheimer's disease dementia; aMCI, amnestic Mild Cognitive Impairment; NC, normal cognition; NIHTB‐OIT, National Institutes of Health Toolbox Odor Identification Test.

^a^ For the linear regression model (Table 3, Equation 1): Adjusted *R* ^2^ = 0.30 (*F*_4,384_ = 40.85, *p* < 0.001). The intercept reflects the mean NIHTB‐OIT scores for NC males at mean age 77.8 years. For the NC vs. aMCI logistic regression model (Table 3, Equation 2): The Akaike Information Criterion of the model was 248.3, with residual deviance of 240.30 (*df* = 227). For the NC vs. ADd logistic regression model (Table 3, Equation 3): The Akaike Information Criterion of the model was 183.69, with residual deviance of 175.69 (*df* = 210). The baseline group was males at mean age 77.8 in both models.

^b^ The present study targeted recruitment of aMCI and ADd individuals, and does not reflect a random sample of the general population. Thus, the computed relative risk for aMCI and ADd with increasing age in our sample may not accurately reflect the prevalence or relative risk of these disorders in the general population.

A second model was evaluated including an interaction term between age and diagnosis. The interaction terms were not significant, so they were not included in the final model (see Table S1). A third model was evaluated excluding the NC aged ≥85 participants, as they were oversampled compared to the *N* = 21 participants age 85 and over in the aMCI and ADd groups. The coefficients of this model were very close to those observed in the model with the NC aged ≥85 participants included (see Table S2). While the effect of sex was significant in the full model, with females scoring higher than males across all diagnosis groups, sex differences were not significant within each diagnosis group, evaluated using Welch two‐sample *t*‐tests.

**FIGURE 1 alz13426-fig-0001:**
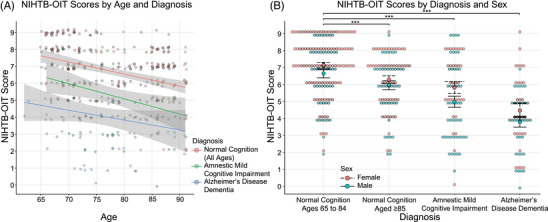
NIHTB‐OIT scores across age and diagnostic categories. (A) Separate regression lines (NIHTB‐OIT score ∼ age) with 95% confidence intervals are fitted for NC (red), aMCI (green), and ADd (blue) diagnostic categories. From the multiple linear regression models, the mean NIHTB‐OIT scores (intercepts) for each group were significantly different, but the rate of NIHTB‐OIT score decline with age (slopes) were not significantly different across diagnostic groups (all interaction terms, *p* > 0.05). Points are jittered for visibility. (B) Dot plots displaying the distribution of NIHTB‐OIT scores across sex and diagnosis categories. Female = red, and Male = blue. Mean NIHTB‐OIT score and standard error of the mean are plotted for each sex and diagnostic group (solid lines, males; dashed, females). While the main effect of sex was significant across all diagnostic groups (linear regression model; *p* < 0.01), it was not significant within each diagnostic group (pairwise Welch two‐sample *t*‐tests, all *p* > 0.05). Mean NIHTB‐OIT scores were significantly different between NC aged 65 to 84 and NC aged ≥85, between NC aged 65 to 84 and aMCI, and between NC aged 65 to 85 and ADd (all *p* < 0.001). ADd, Alzheimer's disease dementia; aMCI, amnestic Mild Cognitive Impairment; NC, normal cognition; NIHTB‐OIT, National Institutes of Health Toolbox Odor Identification Test. ***p < 0.001

Figure 1A shows a scatterplot of NIHTB‐OIT scores versus age, with separate fitted regression lines (NIHTB‐OIT score ∼ age) with 95% CIs for each diagnostic category. While NIHTB‐OIT scores decrease significantly with age, and the mean NIHTB‐OIT scores are significantly different across diagnostic categories (NC all ages, aMCI, and ADd), there is no significant interaction between age and diagnosis (i.e., slopes do not differ significantly across the three diagnostic categories). Figure 1B shows distribution plots for NIHTB‐OIT scores, stratified by sex and diagnostic category (NC aged 65 to 84, NC aged ≥85, aMCI, and ADd).

### 3.2 Utility of the NIHTB‐OIT for detecting aMCI and ADd

To determine whether the NIHTB‐OIT is useful for detecting aMCI and ADd compared to NC aged 65 to 84, we computed ROC curves based on two logistic regression models. The models were fit with male as the baseline group, and centered at the mean age of 77.8 years (see Table 3, Equations (2), (3)). In the *P*(aMCI) model, using Equation (2) shown in Section 2.3, every 1‐point increase in NIHTB‐OIT score was associated with a 25.3% decrease in relative risk of aMCI (*p* < 0.001). Females had a 60.8% decrease in relative risk of aMCI compared to males for a given NIHTB‐OIT score (*p* < 0.005). Each 1‐year increase in age was associated with a 10.8% increase in relative risk for aMCI (*p* < 0.001). In the *P*(ADd) model, based on Equation (3), every 1‐point increase in odor score was associated with a 51.3% decrease in relative risk of ADd (*p* < 0.001). In this model, age and sex were not significantly associated with changes in relative risk of ADd.

**FIGURE 2:**
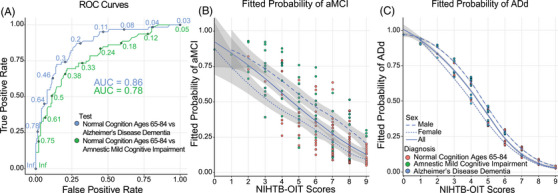
Utility of the NIHTB‐OIT for Detecting aMCI and ADd. (A) Receiver operator characteristic (ROC) curves for classifying aMCI and ADd based on NIHTB‐OIT scores, age, and sex. The calculated area under the curve (AUC) for detecting aMCI was 0.78 (95% CI 0.72 to 0.85), and for detecting ADd was 0.86 (95% CI 0.81 to 0.92). (B,C) Scatter plots of participants’ fitted probability of having aMCI (B) or ADd (C) from the logarithmic regression models described above, plotted against their scores on the NIHTB‐OIT. Loess smoother lines are fitted separately for all participants (solid), female participants (dot‐dashed), and male participants (dashed), with 95% CIs. True diagnoses are indicated by point color (NC, red; aMCI, green; ADd, blue). The legend in panel C applies to both panels B and C. ADd, Alzheimer's disease dementia; aMCI, amnestic Mild Cognitive Impairment; CI, confidence interval; NC, normal cognition; NIHTB‐OIT, National Institutes of Health Toolbox Odor Identification Test

For a threshold of 0.50, the sensitivity and specificity of the *P*(aMCI) model were found to be 49.4% and 88.8%, respectively. The positive predictive value of this model was 69.6%, while the negative predictive value was 77.1%. The calculated AUC of the *P*(aMCI) ROC plot was 0.78 (95% CI 0.72 to 0.85). For a threshold of 0.50, the sensitivity and specificity of the *P*(ADd) model were found to be 56.5% and 89.5%, respectively. The positive predictive value of this model was 68.7%, while the negative predictive value was 83.4%. The calculated AUC of the *P*(ADd) ROC plot was 0.86 (95% CI 0.81 to 0.92). The ROC plots are displayed in[Fig alz13426-fig-0001] Figure 2A. The fitted probability values for having aMCI or ADd are plotted against the NIHTB‐OIT scores for each participant in Figure 2B, color coded by diagnosis, and stratified by sex (indicated by the type of line). For females, an NIHTB‐OIT score of 3 or below has a ≥50% chance of having aMCI, while for males, an NIHTB‐OIT score of 5 or below is has a ≥50% chance of having aMCI. Male odor scores below 5 indicate a ≥50% chance of being classified as ADd compared to NC, while for females odor scores below 4 indicate a ≥50% chance of ADd.

### 3.3 NIHTB‐OIT scores based on AD biomarker presence

**FIGURE 3:**
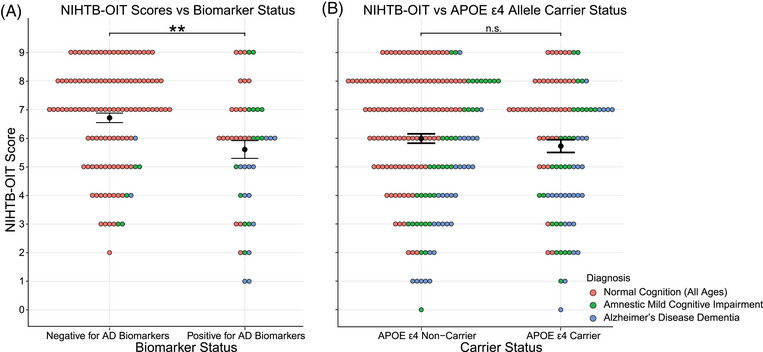
NIHTB‐OIT scores based on AD biomarker presence and *APOE ε4* allele status. (A) Dot plots illustrating the distribution of NIHTB‐OIT scores across AD biomarker groups. Participants positive for AD biomarkers had significantly lower NIHTB‐OIT scores than participants negative for AD biomarkers (*p* < 0.005). (B) Dot plots illustrating the distribution of NIHTB‐OIT scores across *APOE ε*4 allele carrier groups. There was no significant difference in NIHTB‐OIT scores based on *APOE ε*4 allele carrier status. Black points show the mean NIHTB‐OIT score within each group, and error bars reflect the standard error of the mean. AD, Alzheimer's disease; NIHTB‐OIT, National Institutes of Health Toolbox Odor Identification Test. **p < 0.01

The mean ± standard deviation NIHTB‐OIT scores for the biomarker‐positive group and biomarker‐negative group were 5.60 ± 2.15, and 6.71 ± 1.80, respectively. A three‐way ANCOVA was used to evaluate differences in odor scores across biomarker groups while controlling for age and sex. There was a significant main effect of biomarker status (*F*_1,161_ = 7.941, *p* < 0.005), where participants who had a positive AD biomarker test scored lower on the NIHTB‐OIT than participants with a negative AD biomarker test. The main effects of age and sex were also significant (age: *F*_1,161_ = 7.923, *p* < 0.005; sex: *F*_1,161_ = 5.745, *p* < 0.05). The distribution of NIHTB‐OIT scores based on AD biomarker status is shown in Figure 3A.

### 3.4 Differences in NIHTB‐OIT scores based on *APOE ε*4 allele status

The mean ± standard deviation NIHTB‐OIT scores for the *APOE ε*4 allele carriers versus non‐carriers were 5.72 ± 2.21, and 5.99 ± 2.18, respectively. A three‐way ANCOVA was used to evaluate differences in NIHTB‐OIT scores between participants with at least one *APOE ε*4 allele and participants with no *APOE ε*4 alleles, while controlling for age and sex. The main effect of *APOE ε*4 allele status was not significant (*F*_1,271_ = 2.852, n.s.), indicating that NIHTB‐OIT scores did not significantly differ based on *APOE ε*4 allele status. The main effects of age and sex were significant (age: *F*_1,271_ = 16.926, *p* < 0.0001; sex: *F*_1,271_ = 14.611, *p* < 0.0005). The distribution of NIHTB‐OIT scores based on *APOE ε*4 allele carrier status is shown in Figure 3B.

### 3.5 Differences in NIHTB‐OIT scores between NC participants aged 65 to 84 and NC participants aged ≥85

The mean and standard deviation NIHTB‐OIT scores for the two NC age groups are listed in Table 2. A two‐way ANCOVA was used to evaluate differences in NIHTB‐OIT scores between NC participants aged 65 to 84 and NC participants aged ≥85, while controlling for sex. In this model, the main effect of age group was significant (*F*_1,245_ = 12.641, *p* < 0.0001), indicating that participants in the ≥85 years of age group performed significantly worse on the NIHTB‐OIT compared to participants in the 65 to 84 years of age group. The main effect of sex was not significant in this model (*F*_1,245_ = 3.601, n.s.). The distribution of NIHTB‐OIT scores based on NC age group are shown in Figure 1B. We further broke down the mean and standard deviation odor scores across each NC decade: For participants aged 65 to 74 (*N* = 103), the mean ± standard deviation NIHTB‐OIT scores were 7.23 ± 1.67; for participants aged 75 to 84 (*N* = 49), the mean ± standard deviation NIHTB‐OIT scores were 6.43 ± 1.70; and for participants aged 85 to 91 (*N* = 96), the mean ± standard deviation NIHTB‐OIT scores were 6.13 ± 1.70.

## 4 DISCUSSION

We found that mean NIHTB‐OIT scores decrease with age at a similar rate across all diagnostic categories, but were significantly lower for aMCI and ADd participants compared to NC participants, suggesting that olfactory decline may begin earlier in life for those who go on to develop aMCI and ADd. Related findings suggest that olfactory decline may precede aMCI or ADd diagnoses by several years.^12^, ^13^, ^14^ Alternatively, considering the insignificant interaction effects between age and diagnosis on olfactory scores, it is possible that age and disease progression have additive, independent effects on olfaction. Since the NIHTB‐OIT was originally only validated for NC populations up to age 85,^32^ we evaluated test scores for *N* = 96 NC participants aged 85 to 91. We found that NIHTB‐OIT scores for this age group were significantly lower compared to those for NC participants aged 65 to 84, consistent with previous literature suggesting that olfactory ability begins declining slowly in the 40s to 50s and then more rapidly after age 60.^5^, ^7^, ^32^ Olfactory decline is thus associated with increasing age in the absence of aMCI and ADd disease progression.

We found that the NIHTB‐OIT is a useful tool for discriminating between NC participants and participants with prevalent cases of aMCI or ADd, while controlling for sex and age. The calculated ROC AUC values of 0.78 and 0.89 indicate good predictive value for detecting aMCI, and excellent predictive value for detecting ADd, respectively. While the NIHTB‐OIT only correctly flagged about 50% of aMCI cases (sensitivity, 49.4%) and 57% of ADd cases (sensitivity, 56.5%), it had a fairly low rate of false negatives for both diagnostic categories (aMCI specificity, 88.8%; ADd specificity, 89.5%), suggesting that higher NIHTB‐OIT scores are more likely to indicate normal cognition.

Other odor identification tests, including the University of Pennsylvania Smell Identification Test (UPSIT),^44^ the Brief (Cross‐Cultural) Smell Identification Test (B‐SIT),^45^ and the San Diego Odor Identification Test (SDOIT),^46^ have been previously investigated for their utility in detecting prevalent cases of aMCI and ADd. A recent study identified the 10 most predictive odors on the UPSIT for amnestic disorders, and for a cutoff of 70% correct, these 10 odors performed well at identifying prevalent amnestic disorders (aMCI and ADd; 74% sensitivity, 71% specificity) and prevalent ADd (88% sensitivity, 71% specificity) compared to controls.^16^ Another study found that 40% of participants with aMCI and 10% of those with ADd performed at a normosmic threshold (8/12 correct) on the B‐SIT, compared to ∼70% of participants with non‐amnestic MCI or subjective memory complaints.^47^ In longitudinal studies, poor baseline performance on the UPSIT, B‐SIT, and SDOIT tests have been found to predict the incidence of new aMCI and ADd cases over 3‐ to 5‐year time spans.^12^, ^13^, ^14^, ^16^ Conversely, less than 4% of participants with normosmic performance on the B‐SIT declined to dementia over a 4‐year period,^48^ and normosmic performance on the SDOIT had a negative predictive value of 97% for new incidence of cognitive impairment over a 5‐year period.^13^ Future directions of the ARMADA study will include longitudinal analyses to determine whether poor NIHTB‐OIT scores are similarly related to later cognitive impairment.

While the NIHTB‐OIT performs similarly to other olfactory tests in detecting prevalent cases of aMCI and ADd, the NIHTB‐OIT may be more suited for routine clinical use. The SDOIT test, while brief, presents odorants inside small containers impractical to maintain on hand within a clinical setting.^46^ The UPSIT test is much longer (40 odorants) and recent work has suggested that shorter variations, including the B‐SIT, have similar predictive power.^16^, ^49^ However, the NIHTB‐OIT may be more accessible compared to the B‐SIT, which uses word‐based multiple‐choice options in a pencil‐and‐paper format. The NIHTB‐OIT, in contrast, provides picture, word, and verbal response options on a touch‐screen tablet, and may help to control for test‐taker variability in visual, auditory, or lexical ability. One previous study found that a picture‐based odor identification test was more reliable than the word‐based B‐SIT in distinguishing AD participants from controls in a Japanese population.^50^

We found a weak effect of sex on NIHTB‐OIT scores, with females scoring significantly better than males across (but not within) all diagnostic categories. Females have been known to outperform males on tests of olfaction, although usually to a small degree.^51^, ^52^ Our *P*(aMCI) logistic regression model indicates that females have a 60.8% decrease in relative risk for having aMCI compared to males, given a particular age and NIHTB‐OIT score. Scores of 3 and below indicate greater than 50% probability of having aMCI for females, while scores of 5 and below indicate the same for males. While the effect was not significant in the *P*(ADd) logistic regression model, there was still a weak trend for sex. NIHTB‐OIT scores of 5 and below were associated with 50% probability of a diagnosis of ADd for males, while scores of 4 and below indicated the same for females. Thus, clinicians and researchers using the NIHTB‐OIT to evaluate the possible presence of aMCI or ADd should consider sex when interpreting scores.

For a subset of participants, AD biomarker and *APOE* genotype data were available. We found that NIHTB‐OIT scores were significantly lower for participants who had a positive AD biomarker test compared to those who had negative AD biomarker tests, collapsed across available CSF Aß40/42, total tau, or phospho‐tau measures and amyloid‐PET measures. This is consistent with previous findings that the level of AD biomarkers, particularly tau pathology, correlates with performance on odor identification tests.^24^, ^25^, ^53^ Future targeted investigations into the relationships between odor identification scores and amyloid and tau burden will help determine the usefulness of these tests for identifying participants with AD‐specific pathologies.

We determined that NIHTB‐OIT scores did not differ significantly between participants with no *APOE ε*4 alleles, and participants with at least one *APOE ε*4 allele. The *APOE ε*4 allele is associated with an increased lifetime risk of developing aMCI and ADd,^54^ more so for populations with European ancestry compared to populations with African ancestry,^55^ but when comparing *APOE ε*4 allele status and olfactory deficits, there have been mixed results.^26^, ^27^, ^56^, ^57^ Very large sample sizes may be required to detect the incidence of olfactory deficits for *APOE ε*3/*ε*4 heterozygotes.^27^ Within our sample (*N* = 275), only 19 were identified as *APOE ε*4 homozygotes. Our sample size is likely too small to detect such single‐gene effects. Other literature suggests that the effect of *APOE* genotype on olfaction may be independent from its effect on aMCI and ADd risk,^57^ and alternatively, the *ε*4 allele may play a role with regard to which specific brain anatomical regions are likely to be affected by AD pathology, independent from the incidence of the pathology itself.^57^, ^58^, ^59^

We determined that the NIHTB‐OIT has low sensitivity and high specificity for classifying aMCI versus NC, and ADd versus NC. This suggests that a high score on the NIHTB‐OIT indicates a low likelihood of having aMCI or ADd, but a low score does not provide enough evidence for a definitive diagnosis of aMCI or ADd. While diminished olfaction is a common symptom in aMCI and ADd, it is not specific to these diseases, and has also been associated with Parkinson's disease,^60^, ^61^ Huntington's disease,^62^ multiple sclerosis,^63^, ^64^ traumatic brain injuries,^39^ strokes,^40^ and cognitively healthy aging.^32^ The lack of data on smoking status ^65^ is also a limitation of the current study. Additionally, following the COVID‐19 pandemic, more attention must be paid to viral causes of olfactory loss, and potentially associated long‐term effects on neurological health.^66^, ^67^ While the NIHTB‐OIT cannot provide a definitive diagnosis, we suggest that this easily administrable and cost‐effective test may be included in senior patients’ annual physical exams. If a score below 5 is obtained, and other contributing factors and rhinological conditions can be ruled out, the patient may be referred for in‐depth neuropsychological evaluation by a specialist to determine whether other symptoms of cognitive impairment are present.

The generalizability of odor identification tests across different cultural groups must be considered. These tests require familiarity with the presented odors and their correct linguistic descriptors. Many odors are language‐ or culture‐specific. Native speakers perform significantly better on tests developed within their culture compared to nonnative participants.^68^, ^69^, ^70^ We caution that the NIHTB‐OIT may not be accurate in evaluating aMCI risk outside of the United States population. Although the ARMADA study aimed to recruit participants reflecting the racial/ethnic distribution of the U.S. population,^71^ minority populations are still underrepresented in the final sample (e.g., Black participants in the ADd group; and Asian, American Native, or participants of other races and Hispanic/Latino ethnic identity). A future aim of the ARMADA study is to evaluate whether the NIHTB‐OIT can detect aMCI and ADd in two larger cohorts of African American and Spanish‐speaking participants.

In summary, we have provided evidence for the association between NIHTB‐OIT scores and diagnoses across the cognitive aging spectrum, and we have demonstrated that this test has high predictive power for classifying NC versus aMCI, and NC versus ADd.

## 5 CONCLUSION

Olfactory decline is common in aMCI and ADd, and likely occurs earlier on than objective cognitive decline. In the present study, we evaluated performance on the NIHTB‐OIT across healthy controls aged 65 and above, participants with aMCI, and participants with ADd. We found that scores on this test decline with age at a similar rate across all diagnostic groups, but that scores are significantly lower in the aMCI and ADd groups compared to healthy controls. We further found that this test can reliably distinguish healthy controls from participants with aMCI or ADd. Based on our results, we suggest that this quick and cost‐effective test may be included in annual senior wellness exams, and that (in the absence of other rhinological explanations) those with low odor identification scores should be referred for further neuropsychological testing.

## AUTHOR CONTRIBUTIONS

S.L.E.‐C. participated in study design, analysis and interpretation of data, writing the first draft of the report, and writing and editing the report. E.H.H. participated in study design, analysis and interpretation of data, and writing and editing the report. S.W. and R.C.G. participated in study design, interpretation of data, and writing and editing the report. T.K. participated in study design, analysis and interpretation of data, and writing and editing the report.

## CONFLICT OF INTEREST STATEMENT

For all authors, there are no declarations to be made regarding conflicts of interest. Author disclosures are available in the supporting information.

## CONSENT STATEMENT

This study, as part of the multi‐site ARMADA study (Weintraub et al. 2021, Alzheimer's & Dementia), was reviewed and approved by the Northwestern University (lead site) Institutional Review Board (IRB #STU00205290). In addition, each of the participating sites also submitted ARMADA for review to their own IRB and received approval. All human subjects provided informed consent. All research was completed in accordance with the Helsinki Declaration.

## Supporting information

Supporting Information

Supporting Information

Supporting Information
