# Supplementary material for: Evaluation of the NIH Toolbox Odor Identification Test across normal cognition, amnestic mild cognitive impairment, and dementia due to Alzheimer's disease
Source: Alzheimers Dement. 2023 Aug 21;20(1):288–300. doi: 10.1002/alz.13426 (PMC10843554; doi:10.1002/alz.13426)
Supplement: Supplementary file 1 — Supporting Information [file ALZ-20-288-s002.docx]

| **Table S.1. Fitted Linear Regression Model with Interaction Term** | | | | | |
| --- | --- | --- | --- | --- | --- |
| **Multiple Linear Regression Model:**  $NIHTB-OIT Score\sim\beta_{o}+Age\beta_{1}+Sex\beta_{2}+Diagnosis\beta_{3}+Age*Diagnosis\beta_{4}$ | | | | | |
| **Coefficient** | **Estimate** | **Standard Error** | **t-value** | **p-value** | **Interpretation** |
| Intercept | 6.36 | 0.16 | 38.74 | < 0.001 | A male NC participant at the mean age of 77.8 years is predicted to have an NIHTB-OIT Score of 6.36. |
| Age (per year) | -0.07 | 0.014 | -5.05 | < 0.001 | NIHTB-OIT scores are predicted to decrease by 0.07 points for every year increase in age. |
| Sex = Female | 0.54 | 0.19 | 2.88 | 0.0041 | Predicted NIHTB-OIT scores for Females are 0.54 points higher than for Males. |
| Diagnosis = aMCI | -1.30 | 0.24 | -5.53 | < 0.001 | Predicted NIHTB-OIT scores are 1.3 points lower for aMCI participants compared to NC participants. |
| Diagnosis = AD | -2.64 | 0.27 | -9.86 | < 0.001 | Predicted NIHTB-OIT scores are 2.64 points lower for AD participants compared to NC participants. |
| Interaction: Age*Diagnosis=aMCI | -0.02 | 0.03 | -0.74 | 0.46 | The slope between NIHTB-OIT Scores and Age is not significantly different between NC and aMCI participants. |
| Interaction: Age*Diagnosis=AD | 0.01 | 0.04 | 0.37 | 0.72 | The slope between NIHTB-OIT Scores and Age is not significantly different between NC and AD participants. |

‡ Note: Multiple linear regression model comparing NIHTB-OIT scores with age, sex, diagnosis, and interactions between age and diagnosis. Adjusted R^2^ = 0.30 (F_6,382_=27.27, *p* < 0.001). The interaction terms were insignificant, suggesting that the rate of decline in NIHTB-OIT Scores with Age is similar across NC, aMCI, and AD groups.
