# Supplementary material for: Evaluation of the NIH Toolbox Odor Identification Test across normal cognition, amnestic mild cognitive impairment, and dementia due to Alzheimer's disease
Source: Alzheimers Dement. 2023 Aug 21;20(1):288–300. doi: 10.1002/alz.13426 (PMC10843554; doi:10.1002/alz.13426)
Supplement: Supplementary file 2 — Supporting Information [file ALZ-20-288-s001.docx]

| **Table S.2. Fitted Linear Regression Model with NC Ages 85+ Participants Excluded** | | | | | |
| --- | --- | --- | --- | --- | --- |
| **Multiple Linear Regression Model:**  $NIHTB-OIT Score \sim\beta_{o}+Age\beta_{1}+Sex\beta_{2}+Diagnosis\beta_{3}$ | | | | | |
| **Coefficient** | **Estimate** | **Standard Error** | **t-value** | **p-value** | **Interpretation** |
| Intercept  (Baseline Group = Normal Cognition (Ages 65-84)) | 6.10 | 0.23 | 26.91 | < 0.001 | A male NC participant at the mean age of 77.8 years is predicted to have an NIHTB-OIT Score of 6.10. |
| Age (per year) | -0.09 | 0.017 | -5.04 | < 0.001 | NIIHTB-OIT scores are predicted to decrease by 0.09 points for every year increase in age. |
| Sex = Female | 0.63 | 0.22 | 2.85 | < 0.01 | Predicted NIHTB-OIT scores for Females are 0.63 points higher than for Males. |
| Diagnosis = aMCI | -1.08 | 0.27 | -3.98 | < 0.001 | Predicted NIHTB-OIT scores are 1.08 points lower for aMCI participants compared to NC participants. |
| Diagnosis = ADd | -2.50 | 0.28 | -8.86 | < 0.001 | Predicted NIHTB-OIT scores are 2.50 points lower for AD participants compared to NC participants. |
